# Rethinking 1D convolution for lightweight semantic segmentation

**DOI:** 10.3389/fnbot.2023.1119231

**Published:** 2023-02-09

**Authors:** Chunyu Zhang, Fang Xu, Chengdong Wu, Chenglong Xu

**Affiliations:** ^1^Faculty of Robot Science and Engineering, Northeastern University, Shenyang, China; ^2^Shenyang Siasun Robot & Automation Company Ltd., Shenyang, China; ^3^College of Intelligent Systems Science and Engineering, Harbin Engineering University, Harbin, China

**Keywords:** semantic segmentation, lightweight network, 1D convolution, encoder-decoder, feature alignment

## Abstract

Lightweight semantic segmentation promotes the application of semantic segmentation in tiny devices. The existing lightweight semantic segmentation network (LSNet) has the problems of low precision and a large number of parameters. In response to the above problems, we designed a full 1D convolutional LSNet. The tremendous success of this network is attributed to the following three modules: 1D multi-layer space module (1D-MS), 1D multi-layer channel module (1D-MC), and flow alignment module (FA). The 1D-MS and the 1D-MC add global feature extraction operations based on the multi-layer perceptron (MLP) idea. This module uses 1D convolutional coding, which is more flexible than MLP. It increases the global information operation, improving features’ coding ability. The FA module fuses high-level and low-level semantic information, which solves the problem of precision loss caused by the misalignment of features. We designed a 1D-mixer encoder based on the transformer structure. It performed fusion encoding of the feature space information extracted by the 1D-MS module and the channel information extracted by the 1D-MC module. 1D-mixer obtains high-quality encoded features with very few parameters, which is the key to the network’s success. The attention pyramid with FA (AP-FA) uses an AP to decode features and adds a FA module to solve the problem of feature misalignment. Our network requires no pre-training and only needs a 1080Ti GPU for training. It achieved 72.6 mIoU and 95.6 FPS on the Cityscapes dataset and 70.5 mIoU and 122 FPS on the CamVid dataset. We ported the network trained on the ADE2K dataset to mobile devices, and the latency of 224 ms proves the application value of the network on mobile devices. The results on the three datasets prove that the network generalization ability we designed is powerful. Compared to state-of-the-art lightweight semantic segmentation algorithms, our designed network achieves the best balance between segmentation accuracy and parameters. The parameters of LSNet are only 0.62 M, which is currently the network with the highest segmentation accuracy within 1 M parameters.

## 1. Introduction

Semantic segmentation is one of the essential tasks in computer vision, which requires the classification of each pixel of an image. There are many problems in practical applications: application equipment has a small storage capacity and cannot store large-scale networks; equipment needs to complete the calculation of semantic segmentation; reasoning speed needs to be faster to meet actual needs. Based on the above problems, the researchers adjusted the research direction accordingly and proposed lightweight semantic segmentation. The lightweight network has the advantages of fewer parameters, fast operation speed, and segmentation accuracy that meets engineering needs. The earliest lightweight semantic segmentation networks (LSNets) are SegNet (Badrinarayanan et al., 2017), ENet (Paszke et al., 2016), SQNet (Treml et al., 2016), ERFNet (Romera et al., 2017), LinkNet (Chaurasia and Culurciello, 2017), and BiSeNet (Yu et al., 2018). Their segmentation accuracy is around 65 mIoU, and their inference speed is 50 FPS. The segmentation accuracy and inference speed of LSNets that have emerged in recent years have significantly improved. Typical networks include HyperSeg-S (Nirkin et al., 2021), STDC1 (Fan et al., 2021), STDC2, SFNet (Li et al., 2020), and PIDNet (Xu et al., 2022). By reading a lot of semantic segmentation papers, we summarized several directions for lightweight semantic segmentation design: (1) downsampling: reduce the resolution of the input image and reduce the amount of calculation; (2) design efficient convolution: expand the receptive field of convolution, reduce model parameters, and calculations; (3) residual connection: reuse features, improve gradient propagation; (4) design backbone encoding module: standard backbones include ResNet (He et al., 2016), SqueezeNet (Iandola et al., 2016), ShuffleNetV2 (Ma et al., 2018), MobileNet (Howard et al., 2019), and EfficientNet (Tan and Le, 2019).

In this paper, we rethink the application of 1D convolution in lightweight semantic segmentation and design a 1D multi-layer spatial module (1D-MS) and 1D multi-layer channel module (1D-MC). 1D-MS and 1D-MC adopt the idea of the multi-layer perceptron (MLP), simultaneously adds global information. They obtain the best balance in terms of encoding performance and parameters. We also propose a feature alignment module (FA), which solves the problem of feature misalignment on the network, improving segmentation accuracy. Based on the above modules, we designed a 1D-mixer module and an attention pyramid with FA (AP-FA). 1D-mixer adopts the coding structure of the transformer. The first residual connection contains 1D-MSs, and the channel separation operation aims to extract spatial information and reduce the amount of calculation. The second residual connection contains 1D-MCs to facilitate information fusion between channels. The AP-FA module contains an AP and a FA to decode and upsample features. The purpose of our design of the AP-FA module is to fuse multi-scale information, reduce the loss of details, solve the problem of misalignment, and improve the segmentation accuracy. Based on the 1D-mixer and AP-FA modules, we propose an efficient, LSNet consisting entirely of 1D convolutions. The 1D-LSNet network we designed is trained and predicted on only one 1080Ti GPU, and there are no other pre-training operations. On the Cityscapes dataset, a segmentation accuracy of 72.6 mIoU has been achieved, and the number of parameters is 0.62 M. It is currently the lightweight network with the highest segmentation accuracy within 1 M parameters. On the CamVid dataset, our accuracy is 70.5 mIoU, and the inference speed reaches 122 FPS, the model with the highest accuracy among all lightweight networks. On the ADE2K dataset, our network achieves an accuracy of 36.4 mIoU. We transplanted the trained network to the Qualcomm Snapdragon 865 mobile processing device, and the delay time was 224 ms, which met the requirements for mobile devices. Compared with advanced semantic segmentation algorithms, LSNet outperforms the latest lightweight networks regarding segmentation accuracy and parameter balance.

Our contributions can be summarized in the following points:

1. A 1D-MS and a 1D-MC are proposed, which inherit the design idea of MLP and integrate global feature operations. Since this module uses 1D convolution, it is not limited by the input size. This module has the advantages of fewer parameters and strong coding ability.
2. We designed the 1D-mixer module, which adopts the structure of the visual transformer, and combines the 1D-MS module, the 1D-MC module, and the channel separation technology. This module encodes and fuses the feature map along the space and channel direction, which has the advantages of strong encoding ability and few parameters.
3. An AP-FA is proposed. The purpose of the AP is to expand the network receptive field, reduce the loss of details, and improve the segmentation accuracy. At the same time, to solve the loss of accuracy caused by feature misalignment, a FA is proposed for upsampling.
4. Based on the above modules, we designed a LSNet. The network performed well on the Cityscapes and CamVid datasets compared with the advanced LSNet, and it obtained the best balance between accuracy and parameters. The network trained in the ADE2K data set is transplanted to the mobile device, and the delay time is 224 ms, which meets the requirements of the mobile device. The number of parameters of the network we designed is 0.62 M, and the accuracy is the highest among the networks within 1 M parameters.

## 2. Related work

### 2.1. Semantic segmentation

Semantic segmentation (Brempong et al., 2022; Mo et al., 2022; Sheng et al., 2022; Ulku and Akagündüz, 2022) is the vision task of classifying image pixels. FCN (Noh et al., 2015) replaces the FC of the classification network with convolution, enabling the development of end-to-end convolutional networks. Recently, MLP-based networks have shown great potential in object detection and surpassed transformer-based semantic segmentation methods. LEDNet (Wang et al., 2019) is a typical lightweight network. The encoder uses a combination of residual modules and decomposed convolutions, and the decoder uses a simple pyramid structure. The algorithm’s structure conforms to the design principle of lightweight semantic segmentation structure and has the advantages of high segmentation accuracy and few parameters. We summarized the main design ideas of lightweight semantic segmentation through many research papers, mainly multi-scale receptive field fusion, multi-scale semantics, expanding receptive field, strengthening edge features, and obtaining global information.

### 2.2. Attention mechanism

The purpose of the attention mechanism (Guo et al., 2022a,b) is to select features and make reasonable use of computing resources. There are two types of attention mechanisms in semantic segmentation networks, channel attention and spatial attention, which play different roles in the network. Spatial attention focuses on the central region from the perspective of feature space. Channel attention focuses on selecting feature channels and using some channels as the primary encoding object. CBAM (Woo et al., 2018) uses a mixture of typical channels and spatial attention. The most significant advantage of this module is that it has a small number of parameters. It can be seamlessly integrated into any CNN architecture, ignoring additional overhead.

### 2.3. Transformer

The transformer (Han et al., 2022; Khan et al., 2022) was first used in the field of NLP to encode the input sequence. ViT (Dosovitskiy et al., 2020) demonstrates that transformers can also be applied to image classification. ViT treats an image as a sequence and sends it to a transformer layer for classification. ViT-based variants include CPVT (Chu et al., 2021), TNT (Han et al., 2021), and LocalViT (Li et al., 2021), improving image classification accuracy. For semantic segmentation, the core architecture of SETR (Zheng et al., 2021) is still the encoder-decoder structure. However, compared to the traditional CNN-led encoder structure, SETR uses transformer to replace it, but this method could be more efficient. Recently, SegFormer (Xie et al., 2021) designed a novel hierarchical transformer encoder that outputs multi-scale features. It does not require positional encoding, thus avoiding interpolation of positional encodings. SegFormer also has disadvantages: the output resolution is fixed, and the resolution is too low, which affects the detail segmentation.

## 3. Method

### 3.1. 1D-MS and 1D-MC

**FIGURE 1:**
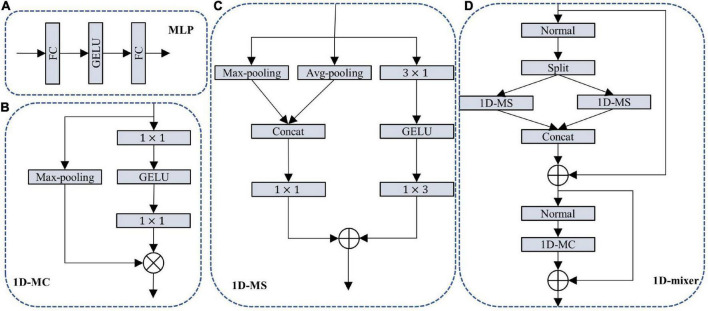
**(A)** Multi-layer perceptron (MLP); **(B)** 1D multi-layer channel module (1D-MC); **(C)** 1D multi-layer space module (1D-MS), and **(D)** 1D-mixer. ⊗ Means pixel multiplication; ⊕ means pixel addition; split means channel separation; concat means channel splicing.

Lightweight semantic segmentation research aims to design a neural network with small parameters and high segmentation accuracy. The current lightweight segmentation network can be divided into two categories: (1) the number of parameters is more than 5 M, and the segmentation accuracy is between 72 and 80 mIoU. The utilization rate of such network parameters is low, and it may be necessary to increase the parameters by about 10 M for every 1 mIoU increase in accuracy. Although the accuracy can meet the application requirements, it deviates from the original intention of lightweight. (2) The number of parameters is below 5 M, and the segmentation accuracy is less than 72 mIoU. The parameter utilization rate of this type of network is high, but the segmentation accuracy could be better. The parameters and segmentation accuracy are challenging to balance. MLP has recently become a new research direction, and its advantages are high segmentation accuracy and a small number of parameters, as shown in Figure 1A. MLP has a fatal shortcoming. It has strict requirements on the input feature size and requires additional feature cropping to be applied to the semantic segmentation network.

Based on the above analysis, we designed a 1D-MS and a 1D-MC. The purpose of our design of these two modules is to inherit the excellent performance of MLP and solve the shortcomings of MLP. The design process is as follows: 1D-MS is divided into a local feature extraction branch and a global information extraction branch, as shown in Figure 1C. The local feature extraction branch adopts the structure of MLP and replaces the fully connected layer with 1D depth separation convolution (convolution kernel size is 3 × 1 and 1 × 3). This not only fits the coding performance of MLP but also solves the problem of input size. Since 1D convolution is used for spatial encoding, there will be decoupling problems in extracting features. To solve this problem, we design the global information extraction branch. This branch uses max-pooling and avg-pooling to obtain global feature information and generates global features through 1 × 1 convolution. The addition of the output features of the two branches not only solves the decoupling problem but also integrates the local and global features to improve the coding performance. The design concept of 1D-MC is similar to that of 1D-MS. As shown in Figure 1B, its channel fusion branch replaces the MLP fully connected layer with 1 × 1 convolution, and the channel selection branch uses the global max-pooling operation. It is worth noting that the number of intermediate feature output channels of our designed channel fusion branch is half the number of input channels. The output of the two branches is multiplied, and 1D-MC not only performs information fusion between channels but also selects feature channels.

The 1D-MS and 1D-MC we designed to have the following advantages: they inherit MLP’s advantages of solid coding ability and fewer parameters; there is no requirement for the input feature size, which is more flexible than MLP; it adds a global feature branch and channel selection branch to improve the overall coding performance of the module.

### 3.2. 1D-mixer module

The design of the encoder is key to the success of the network. Visual transformer is the coding structure that has recently received the most attention and is widely used in object detection and semantic segmentation. The 1D-mixer module we designed uses the transformer architecture. The 1D-mixer module comprises 1D convolution, which extracts and fuses the feature’s spatial and channel information. The 1D-mixer spatial feature encoding part includes the 1D-MS module, channel separation, and residual connection. The role of channel separation is to reduce the number of feature channels and the parameters required for later encoding. 1D-MS is used for encoding in the direction of feature space. This encoding module integrates local and global information and has strong encoding ability. Using residual connections increases the utilization of features and speeds up network training. The 1D-mixer channel information fusion part is composed of 1D-MC and residual connection. This part helps feature information flow between different channels and feature selection along the direction of the channel. The overall structure of the 1D-mixer is shown in Figure 1D, and the specific calculation process is as follows:


(1) S⁢F=C⁢o⁢n⁢c⁢a⁢t⁢(M⁢S⁢(S⁢p⁢l⁢i⁢t⁢(X)))+X

(2) O⁢U⁢T=M⁢C⁢(S⁢A)+S⁢F

Where *X* represents the feature input.*SF* and *OUT* denote spatially encoded features and 1D-mixer encoded output. *Split* means distinct channel separation, *MS* means 1D-MS module, and *MC* is the 1D-MC module. + Means residual connection, and *Concat* means channel splicing.

Our 1D-mixer has the following advantages: (1) it adopts transformer structure to fuse spatial feature information and channel information to improve segmentation accuracy; (2) 1D-MS fuses local and global information of feature space direction with very few parameters; (3) 1D-MC module promotes the flow of feature information in the channel direction and selects effective feature channels; (4) it adopts channel separation operation to reduce model parameters and calculation further.

### 3.3. AP-FA module

**FIGURE 2:**
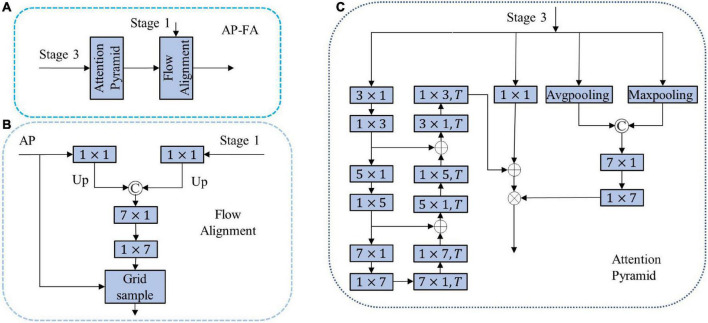
**(A)** Attention pyramid with flow alignment module (AP-FA); **(B)** FA; **(C)** AP. ⊗ Means pixel multiplication; ⊕ means pixel addition; © means channel splicing; *T* means deconvolution.

In order to further extract high-level semantic information and adapt to different tasks, the network usually connects a decoder after the encoder, for which we designed a novel AP-FA, as shown in Figure 2A. The decoder consists of two main parts, one is the attention feature pyramid, and the other is the FA.

#### 3.3.1. Attention pyramid

The AP consists of three branches: 1D pyramid structure, which can further encode features to obtain global information and detailed information; 1 × 1 convolution, which fuses channel information on the output of the encoder; the spatial attention branch acquires features. The spatial position relationship reduces the loss of details. The specific operation process is shown in Equation (3).


(3) $$O\,U\,T=\left[C_{1\times 1}\,\left(X\right)+P\,\left(X\right)\right]\times S\,A\,\left(X\right)$$

Where *X* and *OUT* represent the output feature of the Stage 3 and output of AP, *P* is the pyramid structure, *C*_1 × 1_ is 1 × 1 convolution, *SA* is spatial attention, +represents the addition of corresponding elements, and represents the multiplication of corresponding elements. In the pyramid structure, the convolution and deconvolution of the depth-wise convolution kernel sizes we use are (3 × 1, 5 × 1, and 7 × 1). There are two main reasons for using decomposed convolution here. One is that banded convolution meets the needs of lightweight networks, and the second is that most detected targets are banded. Therefore, using banded convolution is helpful for feature decoding. In the spatial attention branch, two kinds of pooling are used to obtain global information from multiple aspects and are encoded by 1 × 7 and 7 × 1 convolutions. 1 × 7 and 7 × 1 large convolutions can extract spatial features very well. AP related details are shown in Figure 2C.

#### 3.3.2. Flow alignment

Ordinary upsampling will cause the problem of feature misalignment, resulting in decreased segmentation accuracy. We design a FA to restore the resolution and solve the misalignment problem by predicting the flow field inside the network. The specific process is shown in Figure 2B. The input of FA is the output feature (*F*_1_) of Stage 1 and the output feature map (*D*) of AP. The feature map is obtained through a 1 × 1 convolutional layer to obtain a feature map with a channel number of 1. The resulting feature map is upsampled to ensure that the resolution of the two features is equal to the resolution of the input image. We concatenate them together and feed the concatenated feature maps into 7 × 1 and 1 × 7 concatenated convolutional networks. The above steps can be written as follows:


(4) $$o\,f\,f\,s\,e\,t=C\,o\,n\,v\,\left(U\,\left(C_{1\times 1}\,\left(F_{1},D\right)\right)\right)$$

Among them, *U* represents the connection and upsampling operation, *C*_1 × 1_ is a 1 × 1 convolutional layer, *Conv* is a series network of 7 × 1 and 1 × 7. *offset* is the offset required for bilinear interpolation. We normalize *offset* and sum it with the grid to generate an upsampling index. The features output by the AP is upsampled through the grid sample operation. The FA we designed combines high-level semantic features and low-level structural features to solve the problem of feature misalignment perfectly.

The AP-FA structure we designed has the following advantages: first, the pyramid structure is used to extract features, and the purpose is to expand the network receptive field and obtain more decoding features; second, the spatial attention structure suppresses unnecessary information, highlights important information, and improves segmentation precision. Third, the FA method solves the misalignment problem when bilinear interpolation is used for upsampling and improving segmentation accuracy.

### 3.4. Network architecture

**FIGURE 3:**
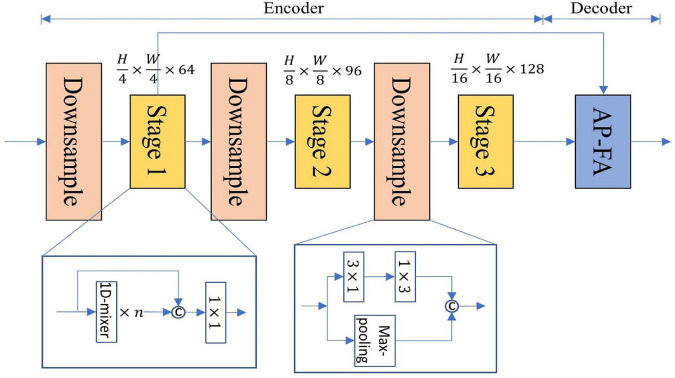
The overall network architecture of lightweight semantic segmentation network (LSNet).

Figure 3 is a structural diagram of LSNet, which uses an asymmetric encoder-decoder structure. The details of the specific design are shown in Table 1. The encoding part uses three stages to encode different resolution features, and the number of 1D-mixer in each stage is 3, 3, 21. The input resolutions of each stage are $\left(H_{\frac{1}{4}}\times W_{\frac{1}{4}},H_{\frac{1}{8}}\times W_{\frac{1}{8}},a\,n\,d\,H_{1/16}\times W_{1/16}\right)$, where *H* and *W* are the height and width of the input image, respectively. The downsampling is 3 × 1 and 1 × 3 convolution concatenation, the step size is 2, and the max-pooling output is spliced simultaneously.

**TABLE 1:** The detailed architecture of lightweight semantic segmentation network (LSNet).

Stage	Type	Channel	Output size
Encoder	Downsampling	64	512 ×256
	1D-mixer ×3	64	512 ×256
	Downsampling	96	256 ×128
	1D-mixer ×3	96	256 ×128
	Downsampling	128	128 ×64
	1D-mixer ×21	128	128 ×64
Decoder	AP-FA	*C*	1,024 ×512

“Channel” denotes the number of output feature maps and “*C*” is the number of classes. “Output size” denotes the output size with an input size of 1,024 × 512.

The input of the AP-FA decoder comes from the feature maps of Stage 1 and Stage 3, and the final scene parsing is performed through the attention feature pyramid and the FA. Much lightweight semantic segmentation ignores the decoder in order to reduce network parameters. A dense decoder can help improve segmentation accuracy without generating too many parameters. Many lightweight networks use three-stage encoders to cause the network’s receptive field to be too small, and bilinear interpolation has problems with upsampling misalignment. Aiming at the problem of the decrease in segmentation accuracy caused by the above, we designed the AP module to expand the network receptive field and increase the global information. We design a FA to restore feature resolution and improve segmentation accuracy.

## 4. Experiments

### 4.1. Datasets and implementation details

#### 4.1.1. Cityscapes

Cityscapes (Cordts et al., 2016) is an urban scene parsing dataset commonly used for semantic segmentation training. It contains street scenes in multiple cities and 5,000 car-driving images collected from the driver’s perspective. This network splits the dataset into 2,975, 500, and 1,525 for training, validation, and testing. We select 19 of these semantic categories for training. We convert the resolution of the original image from 2, 048 × 1, 024 to 1, 024 × 512 to improve the running speed. We do not introduce additional pre-training during training.

#### 4.1.2. CamVid

CamVid (Brostow et al., 2008) contains 701 street view images, of which 367 are used for training, 101 for validation, and 233 for testing. The data set semantically annotates 32 objects in the picture, and we only train 11 semantic objects. We reduce the resolution of the original image from 960 × 720 to 480 × 360 to improve the inference speed.

#### 4.1.3. ADE2K

ADE2K contains 25,000 pictures, and the resolution of each picture is not uniform. We unified the size of the pictures to 512 × 512 to facilitate model training. The training set contains 20,000 images, the validation set contains 2,000 images, and the test set contains 3,000 images.

#### 4.1.4. Implementation details

All our experiments are run on a 1080Ti GPU. PyTorch 1.7, CUDA 9.0, cuDNN 8.0, and Anaconda environment are specific configurations. For fairness, we adopted the training configuration widely used by everyone. The details are as follows: the stochastic gradient descent method (SGD) is used, the loss function is the cross-entropy, and the learning rate update strategy uses “poly.” The input image is randomly cropped, inverted, and scaled, and the scaling range is 0.75−2. The initial learning rate of training Cityscapes is *1e-2*, the weight decay is *5e-4*, the cropping size is 512 × 512, and the number of input images is eight. The initial learning rate of training. Initial learning rate of CamVid is *1e-3*, the weight decay is *5e-4*, the cropping size is 480 × 360, and the number of input images is 16. Initial learning rate of ADE20K is 1.2*e*−4, the weight decay is *1e-2*, the cropping size is 512 × 512, and the number of input images is eight.

### 4.2. Ablation study

#### 4.2.1. Ablation study for 1D-mixer module

##### 4.2.1.1. Ablation for typical module

**FIGURE 4:**
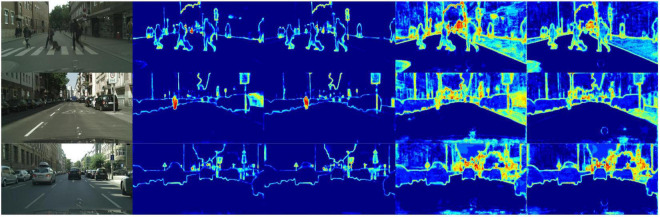
The lightweight semantic segmentation network (LSNet) feature visualization. The picture from left to right is: the original image, the encoder feature map using DAB, the encoder feature map using 1D-mixer, the network output feature map using DAB, and the network output feature map using 1D-mixer.

We compare LEDNet’s (Wang et al., 2019) encoding structure SS-nbt, DABNet’s (Li G. et al., 2019) encoding structure DAB, and CGNet’s (Wu et al., 2020) CG encoder with our designed 1D-mixer. We trained on the Cityscapes dataset, replacing the classic module 1D-mixer in the LSNet network. As shown in Table 2, the LSNet network with the CG module has minor parameters, but the accuracy is 8.2 mIoU lower than the network with 1D-mixer. The parameters of the remaining two modules are more than three times that of the 1D-mixer, and the accuracy is also lower than the modules we designed. Figure 4 is a feature visualization diagram of the LSNet network using the 1D-mixer module and the DAB module. Through the above comparative analysis, the 1D-mixer we designed outperforms the classic lightweight encoding modules in feature extraction and parameters.

**TABLE 2:** Ablation study results of 1D-mixer module.

Type	Model	mIoU (%)	Params (M)
Baseline	LSNet	72.6	0.62
Ablation for typical module	SS-nbt	69.8	2.52
	DAB	71.2	2.15
	CG	64.4	0.48
Ablation for depth	3, 9	65.6	0.40
	3, 12	67.2	0.46
	6, 12	67.4	0.49
	3, 15	68.8	0.51
	6, 15	67.5	0.54
	3, 18	70.2	0.57
	3, 24	72.3	0.67
Ablation for 1D-MS	3 × 3	70.9	2.31
	3 × 3 depth-wise	69.8	0.64
Ablation for 1D-MC	1 × 1	71.4	0.62

##### 4.2.1.2. Ablation for depth

The LSNet network contains three encoding stages, and the number of layers set in the first stage is three, which is consistent with the design of most classic lightweight networks. We experimented with the number of modules in the second and third stages of the network, hoping to find a suitable number of layers to achieve a certain balance between the segmentation accuracy and parameters of the network. As shown in Table 2, the segmentation accuracy and model parameters increase as the number of network layers increases. When the network exceeds a certain number of layers, the segmentation accuracy does not increase. We denote the number of encoders in the second stage by *N*, and *M* is the number of encoders in the third stage. When *M* = 12, the network accuracy of *N* = 3 is 0.2 mIoU higher than that of *N* = 6. The network accuracy is the highest when *N* = 3 and *M* = 21. After the above analysis, we set to *N* = 3 and *M* = 21 in Stage 2 and 3.

##### 4.2.1.3. Ablation for 1D-MS

According to the idea of MLP and global information fusion technology, we designed the 1D-MS module. The 1D-MS module plays the role of spatial feature extraction in the encoder. To explore the superiority of our designed 1D-MS block encoding, we replace 1D-MS with 3 × 3 convolution and 3 × 3 depth-wise convolution. As shown in Table 2, 3 × 3 depth-wise convolution has the same parameters as our designed 1D-MS module, but the accuracy drops by 2.8 mIoU. The 3 × 3 convolution is not as powerful as the 1D-MS module in terms of accuracy and parameters. The above experimental results prove that the encoding effect of our designed 1D-MS exceeds that of ordinary convolution.

##### 4.2.1.4. Ablation for 1D-MC

Information fusion between channels can improve network accuracy. We design the 1D-MC module, adopting the ideas of MLP and channel selection. Ordinary channel information fusion uses 1 × 1 convolution, and here we compare 1D-MC with it. As shown in Table 2, 1D-mixer with 1 × 1 convolution has the same parameters as 1D-MC, but the accuracy is reduced by 1.2 mIoU. It can be seen from the experiments that efficient channel information fusion can improve segmentation accuracy, and our designed 1D-MC is more suitable for channel information fusion than 1 × 1 convolution.

### 4.2.2. Ablation study for AP-FA module

#### 4.2.2.1 Ablation study for AP

Attention pyramid can fuse multi-scale information and perform feature screening simultaneously to improve network accuracy. We conduct ablation experiments on the AP structure, replacing the AP module with 1 × 1 convolution. As can be seen from Table 3, the accuracy of the network without the AP module drops by 2.1 mIoU. From the experiments, it can be seen that adequately designing the decoder can improve network accuracy.

**TABLE 3:** Ablation study results of attention pyramid with flow alignment module (AP-FA) module.

Type	Model	mIoU (%)	Params (M)
Baseline	LSNet	72.6	0.62
Ablation for AP	1 ×1	70.5	0.59
Ablation for attention	–	72.2	0.62
Ablation for feature pyramid	–	70.9	0.59
	333	71.9	0.61
	235	72.0	0.61
	135	71.5	0.61
	3,579	72.5	0.62
Ablation for FA	Bilinear interpolation	70.8	0.62

#### 4.2.2.2 Ablation study for attention

We introduced spatial attention in AP-FA; the purpose is to extract the overall structural features of the feature map and filter the features to improve the segmentation accuracy. To demonstrate the role of spatial attention in the decoder, we compare LSNet with LSNet without attention. Table 3 shows that the accuracy of the network without spatial attention drops by 0.4 mIoU. This test shows that our spatial attention branch can improve network segmentation accuracy.

#### 4.2.2.3 Ablation study for feature pyramid

We use 3 × 1, 5 × 1, and 7 × 1 convolution and deconvolution to form a feature pyramid, the purpose of which is to increase the depth of the network and integrate contextual scale information. We designed five sets of 1D convolution, and the convolution kernel sizes are ((3, 3, 3) , (1, 3, 5) , (2, 3, 5) , (3, 5, 7) , (3, 5, 7, 9)). In order to further prove the value of the pyramid, we designed LSNet to remove the pyramid structure. It can be seen from Table 3 that introducing the pyramid structure can increase 1.7 mIoU. Comparing the experimental results of the LSNet network using these five sets of convolution kernels, the segmentation accuracy of the convolution kernel (3, 5, 7) is the highest, and it is proved that further increasing the depth of the pyramid has little effect on the segmentation accuracy.

#### 4.2.2.4 Ablation study for FA

Since the output resolution of the encoder is smaller than the resolution of the original image, bilinear interpolation is usually used to restore the feature resolution at the end of the network. There is a problem of feature misalignment in bilinear upsampling, which affects the segmentation accuracy. We design a FA in the decoder to solve this problem. We compared bilinear interpolation with FA, and the specific results are shown in Table 3. The FA we designed is 1.8 mIoU higher than the bilinear interpolation algorithm, which shows that the design of the alignment module is effective.

### 4.3. Evaluation results on Cityscapes

**FIGURE 5:**
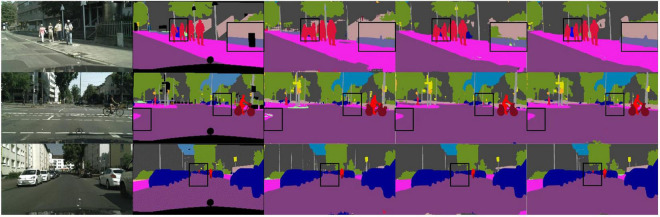
Some visual comparisons on the Cityscapes validation set. From left to right are input images, ground truth, predicted results from LEDNet, DABNet, and our lightweight semantic segmentation network (LSNet).

We designed an LSNet with a parameter of 0.62 M, an inference speed of 95.6 FPS, and a segmentation accuracy of 72.6 mIoU on a 1080Ti. It can be seen from Table 4 that the network we designed has the highest accuracy among the networks with less than 1 M parameters. Under the same experimental conditions of 1080Ti, the network we designed is 69.6 FPS faster than SFNet, and the parameters are also reduced by 12.25 M. From the balance of network parameters and segmentation accuracy, the parameter expression ability of the LSNet we designed is better than that of SFNet. For PIDNet, the segmentation accuracy is 6.2 mIoU higher than LSNet, but 6.98 M increases the number of parameters. From the perspective of accuracy and parameter balance, the parameters of PIDNet are 11 times that of LSNet, but the accuracy increases very little. The network we designed has a better balance. It is worth noting that the resolution of our network input is 1, 024 × 512, and the resolution of PIDNet and SFNet input is 2, 048 × 1, 024, which is an important reason why their accuracy is higher than our network. We compare the visualization results of DABNet, LEDNet, and our designed LSNet, as shown in Figure 5.

**TABLE 4:** Evaluation results of our lightweight semantic segmentation network (LSNet) and other state-of-the-art real-time semantic segmentation models on the Cityscapes test set.

Model	Input size	Pre-train	GPU	mIoU (%)	FPS	Params (M)
SegNet (Badrinarayanan et al., 2017)	640 x 360	ImageNet	TitanX	57	16.7	29.5
ENet (Paszke et al., 2016)	640 x 360	No	TitanX	58.3	135.4	0.4
ICNet (Zhao et al., 2018)	1,024 x 2,048	ImageNet	TitanX	69.5	30.3	26.5
ERFNet (Romera et al., 2017)	512 x 1,024	No	TitanX	68	41.7	2.1
ESPNet (Mehta et al., 2018)	512 x 1,024	No	TitanX	60.3	112	2.1
BiSeNet (Yu et al., 2018)	768 x 1,536	ImageNet	TitanX	68.4	72.3	5.8
Fast-SCNN (Poudel et al., 2019)	1,024 x 2,408	ImageNet	TitanX	68	123.5	1.11
ESPNetV2 (Mehta et al., 2019)	512 x 1,024	No	TitanX	66.2	67	1.25
DFANet (Li H. et al., 2019)	512 x 1,024	ImageNet	TitanX	70.3	160	7.8
LEDNet (Wang et al., 2019)	512 x 1,024	No	1080Ti	69.2	71	0.94
ESNet (Lyu et al., 2019)	512 x 1,024	No	1080Ti	69.1	63	1.66
DABNet (Li G. et al., 2019)	512 x 1,024	No	1080Ti	70.1	104	0.76
FDDWNet (Liu et al., 2020)	512 x 1,024	No	2080Ti	71.5	60	0.8
DDPNet (Yang et al., 2020)	768 x 1,536	No	1080Ti	74.0	85.4	2.52
LEANet (Zhang et al., 2022)	512 x 1,024	No	1080Ti	71.9	77.3	0.74
SFNet (Li et al., 2020)	1,024 x 2,048	No	1080Ti	78.9	26	12.87
PIDNet-S (Xu et al., 2022)	1,024 x 2,048	No	3,090	78.8	93.2	7.6
LSNet (Our)	512 x 1,024	No	1080Ti	72.6	95.6	0.62

### 4.4. Evaluation results on CamVid

Table 5 compares the performance of LSNet on the CamVid dataset with other models. The network we designed has the highest accuracy in the current LSNet, which is 3 mIoU higher than LEANet (Zhang et al., 2022). Without any pre-training, the LSNet network has an accuracy of 70.5 mIoU and a speed of 122 FPS. Our training is only done on a 1080Ti GPU, and the input resolution uses low-resolution images. Unlike most real-time semantic segmentation models, LSNet has apparent advantages: fewer parameters and high segmentation accuracy. Whether it is the Cityscapes or CamVid dataset, our LSNet has excellent performance and strong robustness.

**TABLE 5:** Evaluation results of our lightweight semantic segmentation network (LSNet) and other state-of-the-art real-time semantic segmentation models on the CamVid test set.

Model	Input size	Pre-train	GPU	mIoU (%)	FPS	Params (M)
SegNet (Badrinarayanan et al., 2017)	360 x 480	ImageNet	TitanX	55.6	–	29.5
ENet (Paszke et al., 2016)	360 x 480	No	TitanX	51.3	–	0.4
ICNet (Zhao et al., 2018)	720 x 960	ImageNet	TitanX	67.1	27.8	26.5
CGNet (Wu et al., 2020)	360 x 480	No	2 x V100	65.6	–	0.5
BiSeNet (Yu et al., 2018)	720 x 960	ImageNet	TitanX	65.6	175	5.8
BiSeNetV2 (Yu et al., 2021)	720 x 960	ImageNet	TitanX	68.7	124.5	49.0
DFANet (Li H. et al., 2019)	720 x 960	ImageNet	TitanX	64.7	120	7.8
DABNet (Li G. et al., 2019)	360 x 480	No	1080Ti	66.2	124.4	0.76
LRNNet (Jiang et al., 2020)	360 x 480	No	1080Ti	67.6	83	0.67
DDPNet (Yang et al., 2020)	360 x 480	No	1080Ti	67.3	–	1.1
LEANet (Zhang et al., 2022)	360 x 480	No	1080Ti	67.5	98.6	0.74
LSNet (Our)	360 x 480	No	1080Ti	70.5	122	0.62

### 4.5. Evaluation results on ADE20K

We train all networks on the server and use TNN to port the trained networks to mobile devices. The LSNet we designed and the advanced algorithm are compared on the validation dataset on ADE20K, and the latency (ms) is tested on a mobile device with a single Qualcomm Snapdragon 865 processor. The experimental results are shown in Table 6. FCN-8s, PSPNet (Zhao et al., 2017), R-ASPP (Sandler et al., 2018), and Lite-ASPP (Chen et al., 2018), use MobileV2 as the encoder. LR-ASPP (Howard et al., 2019) uses MoblieV3 as the encoder. We also compare with the advanced lightweight transformer algorithm, where SegFormer uses MiT-B0 as the encoder, and Semantic FPN (Kirillov et al., 2019) uses ConvMLP-S as the encoder. As can be seen from Table 6, LSNet and Lite-ASPP are comparable in latency and segmentation accuracy. However, LSNet has more advantages in calculation amount (GFLOPs) and parameter amount. This experiment proves that the network we designed can be used on mobile devices, and the calculation amount, parameter amount, and segmentation accuracy achieve the best balance.

**TABLE 6:** Results of typical networks on the ADE20K validation set.

Model	Params (M)	FLOPs (G)	mIoU (%)	Latency (ms)
FCN-8s (Noh et al., 2015)	9.8	39.6	19.7	1,015
PSPNet (Zhao et al., 2017)	13.7	52.2	29.6	1,065
R-ASPP (Sandler et al., 2018)	2.2	2.8	32.0	177
Lite-ASPP (Chen et al., 2018)	2.9	4.4	36.6	235
LR-ASPP (Howard et al., 2019)	3.2	2.0	33.1	126
SegFormer (Xie et al., 2021)	3.8	8.4	37.4	770
Semantic FPN (Kirillov et al., 2019)	12.8	33.8	35.8	777
LSNet (Our)	0.65	3.8	36.4	224

All networks are trained on the server and ported to mobile devices through TNN. Latency and GFLOPs calculations take 512 × 512 resolution images as input. Latency measured based on a single Qualcomm Snapdragon 865 processor. All results are evaluated using a single thread.

## 5. Conclusion

In this paper, we designed a LSNet. The network’s success is attributed to the combination design of 1D convolution. Our network transforms the MLP idea into a 1D convolution multi-layer combination, which solves problems where MLP is challenging to apply in semantic segmentation. At the same time, the design of the decoder increases the network’s depth, solves the misalignment of upsampling, and further improves the accuracy of network segmentation. Experimental results show that our designed network achieves the best balance of accuracy and parameters, surpassing the current state-of-the-art lightweight language segmentation network. This paper shows that the proper use of multi-layer 1D convolution is more suitable for semantic segmentation than MLP. Clever decoder design is also an essential part of improving segmentation accuracy. We hope this paper encourages researchers to investigate the potential of 1D convolutions further.

## Data availability statement

The original contributions presented in this study are included in this article/supplementary material, further inquiries can be directed to the corresponding author.

## Author contributions

CZ, FX, CW, and CX performed the material preparation, data collection, and analysis. CZ wrote the first draft of the manuscript. All authors have study conception and design, commented on previous versions of the manuscript, read, and approved the final manuscript.
